# Surgical management of primary undifferentiated pleomorphic sarcoma of the rectum: a case report and review of the literature

**DOI:** 10.1186/s12957-022-02671-6

**Published:** 2022-06-13

**Authors:** Keita Kodera, Masato Hoshino, Sumika Takahashi, Suguru Hidaka, Momoko Kogo, Ryosuke Hashizume, Tomonori Imakita, Mamoru Ishiyama, Masaichi Ogawa, Ken Eto

**Affiliations:** 1 Department of Surgery, Katsushika Medical Center, The Jikei University School of Medicine, 6-41-2 Aoto, Katsushika-Ku, Tokyo, 125-8506 Japan; 2 Department of Gastrointestinal Surgery, The Jikei University School of Medicine, 3-25-8 Nishishimbashi, Minato-ku, Tokyo, 105-8461 Japan

**Keywords:** Undifferentiated pleomorphic sarcoma (UPS), Malignant fibrous histiocytoma (MFH), Rectum, Surgery, Radiotherapy, Chemotherapy

## Abstract

**Background:**

Undifferentiated pleomorphic sarcoma (UPS) is a malignant soft tissue tumor that has been reclassified from malignant fibrous histiocytoma with the development of the pathological diagnosis. It principally occurs in the extremities but rarely occurs in the rectum. We herein report a rare case of UPS arising in the rectum.

**Case presentation:**

A 85-year-old woman was referred to our hospital with a complaint of anal pain, which had persisted for several months. Computed tomography (CT) showed a 53 × 58 × 75 mm mass on the left side of the rectum. Colonoscopy revealed a submucosal elevation in the rectum without any exposure of the tumor to the surface. Contrast-enhanced CT and magnetic resonance imaging revealed an 80-mm mass that originated in the rectal muscular propria, and we suspected a gastrointestinal stromal tumor. No lymph node metastasis or distant metastasis was observed. We performed a laparoscopic Hartmann’s operation. Intraoperatively, severe adhesion around the tumor caused tumor injury and right ureteral dissection. Thus, laparoscopic right ureteral anastomosis and ureteral stenting were additionally performed. The operation time was 6 h and 3 min, and the estimated blood loss was small. The patient was discharged without complications 25 days after surgery. A pathological examination showed that the tumor was composed of highly heterogeneous cells with no specific differentiation traits, leading to a diagnosis of UPS. Contrast-enhanced CT performed 2 months after surgery showed bilateral pelvic lymph node enlargement, which indicated recurrence. Considering the patient’s age, we performed radiotherapy (50 Gy/25 Fr targeting the pelvic region). At present, 16 months have passed since the completion of radiotherapy. Contrast-enhanced CT shows that the recurrent lymph nodes have disappeared, and no new distant metastasis has been observed.

**Conclusions:**

We reported a case of UPS arising in the rectum. The surgical procedure and indication of preoperative therapy should be carefully selected because complete removal of the tumor is desirable in UPS.

## Background

Undifferentiated pleomorphic sarcoma (UPS) is a malignant soft tissue tumor that was reclassified from malignant fibrous histiocytoma (MFH) by the World Health Organization (WHO) in 2002 and 2013 due to changes in the pathologic diagnosis [1]. Although the cells of origin of UPS have not been identified, it can occur anywhere in the body, most commonly in the extremities, but can also occur in the retroperitoneal space [2]. The occurrence of UPS in the rectum is very rare and has only been reported in a few cases. We herein report a rare case of UPS arising in the rectum of an adult female.

## Case presentation

**Fig. 1:**
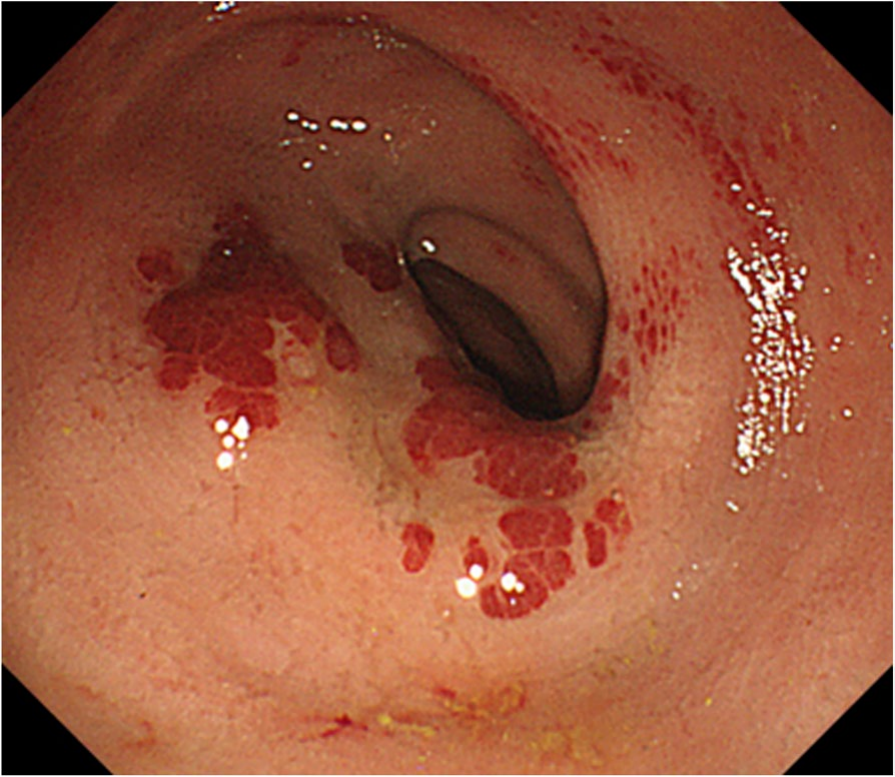
Colonoscopy showed submucosal elevation of the middle and lower rectum with mucosal surface erythema, but no exposure of the tumor to the mucosal surface

**Fig. 2:**
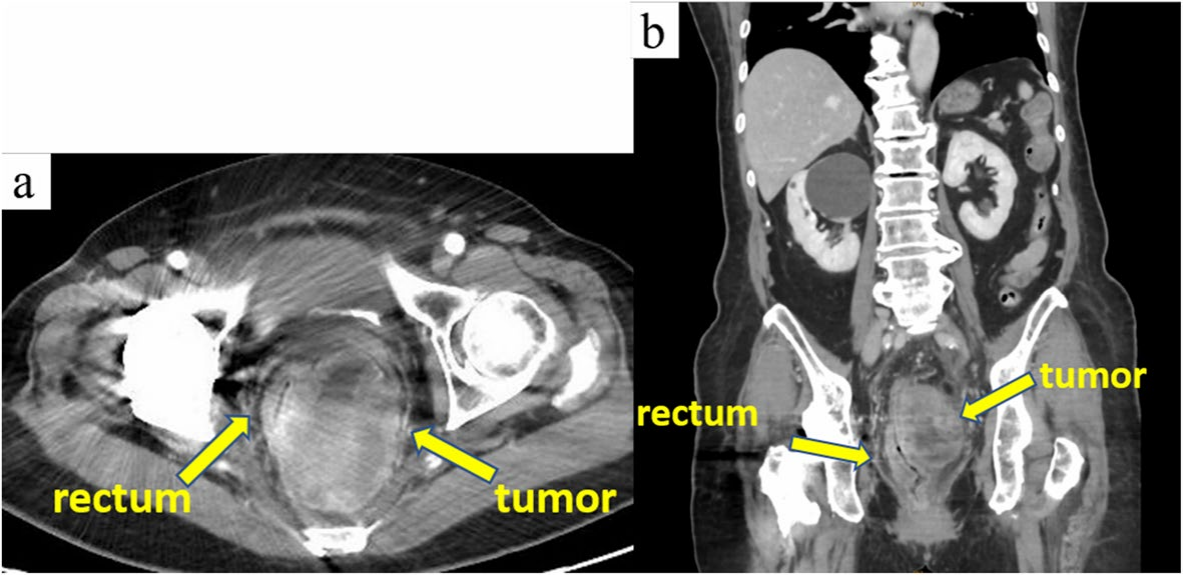
Contrast-enhanced computed tomography revealed a 53 × 58 × 75 mm mass with well-defined boundaries and a heterogeneous contrast effect on the left side of the rectum. Fluid accumulation was observed in the center of the mass, which suggested necrotic tissue

**Fig. 3:**
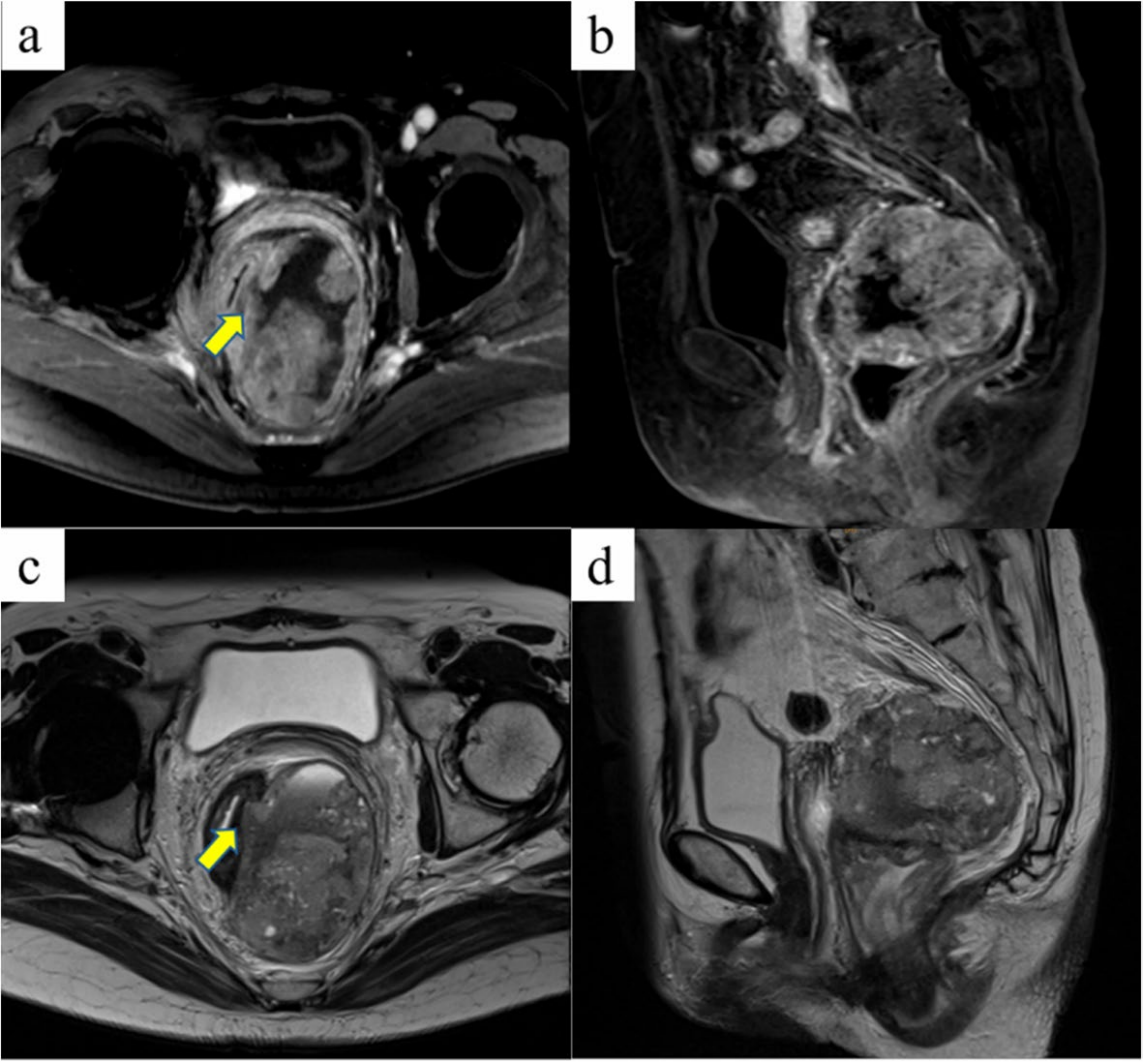
Contrast-enhanced pelvic magnetic resonance images. Coronal fat-suppressed T1-weighted (**a**), sagittal fat-suppressed T1-weighted (**b**), coronal T2-weighted (**c**), and sagittal T2-weighted images (**d**) are presented. The tumor was continuous with the muscular propria on the left side of the rectum (indicated by an arrow)

In 2020, an 85-year-old woman presented to her family doctor with a complaint of anal pain that had persisted for months. Abdominal computed tomography (CT) showed a 53 × 58 × 75 mm mass on the left side of the rectum. She was admitted to our hospital for further examination and treatment. She had a medical history of open appendicectomy for appendicitis, open total hysterectomy for uterine fibroids, femoral head replacement for right femoral neck fracture, hypertension, and dyslipidemia. None of her family had a clear history of cancer. A hematological examination showed no elevation in tumor or inflammation markers, with the exception of CA125 (55 U/mL). On visual examination, there were no obvious abnormalities of the anus. On digital anorectal examination, an elastic hard mass was palpated on the left side of the rectum, and tenderness was present in the same region. Colonoscopy showed submucosal elevation and reddening of the mucosal surface in the central and lower rectum, but no obvious exposure of the tumor to the mucosal surface (Fig. 1). Contrast-enhanced computed tomography (CT) showed a 53 × 58 × 75 mm mass lesion on the left side of the rectum with well-defined margins and heterogeneous contrast enhancement. Fluid accumulation was observed in the center of the mass, which suggested necrotic tissue (Fig. 2 a and b). No lymph node metastasis or distant metastasis was observed. Contrast-enhanced pelvic magnetic resonance imaging (MRI) revealed that the tumor was continuous with the muscular propria on the left side of the rectum (Fig. 3 a, b, c, and d). According to the imaging findings, we suspected that the tumor was a gastrointestinal stromal tumor, and we planned to obtain a pathological diagnosis by endoscopic ultrasound fine-needle aspiration. However, since the anal pain worsened rapidly and was uncontrollable despite the introduction of opioids, we decided to perform early surgery without a preoperative pathological diagnosis. A laparoscopic Hartmann’s operation was planned.

**Fig. 4:**
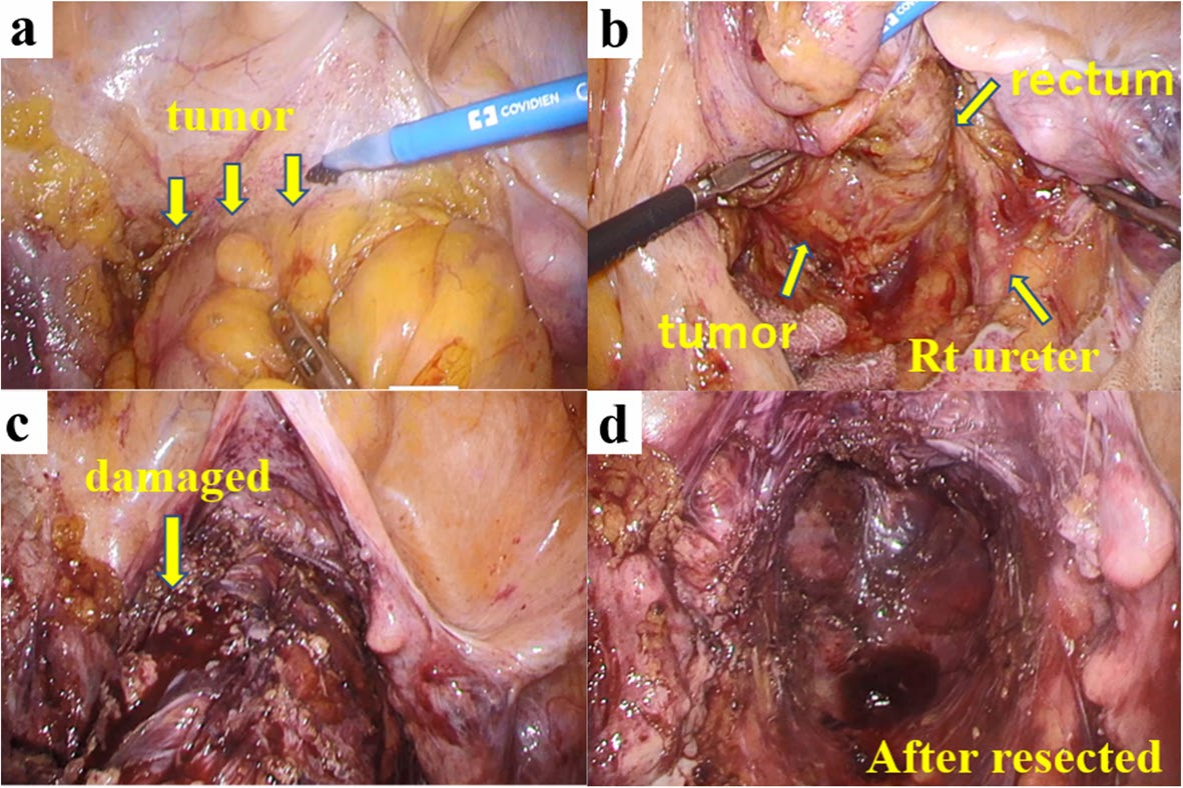
Intraoperative findings. The tumor was located caudal to the peritoneal reversal and firmly adherent to the bladder and the vagina (**a**). The tumor was so large that the rectum was pushed to the right side (**b**). The tumor was damaged in the region, causing the leakage of dark red tumor contents (**c**). The rectum and tumor were removed by a Hartmann operation (**d**)

After administering general anesthesia, the patient was placed in the lithotomy position and underwent laparoscopic surgery using 5 ports. As in rectal surgery, the retroperitoneum was dissected caudally from the promontorium using a medial approach, and the rectal mesentery was mobilized. The tumor was located caudal to the peritoneal reversal and occupied the pelvic cavity (Fig. 4a). The tumor was so large that the rectum was pushed to the right side, and the border was unclear due to the surrounding inflammation (Fig. 4b).

Therefore, dissection of the right side of the tumor was so difficult that the right ureter was misidentified and accidentally separated. The ventral side of the tumor was firmly adherent to the bladder, and the tumor was damaged at the region, causing the leakage of dark red tumor contents (Fig. 4c). The lower rectum with 6 cm of the anal verge was dissected using a linear stapler (Signia™, Medtronic, Tokyo, Japan) (Fig. 4d). The rectum was elevated outside the body, and the sigmoid colon was resected at a distance of 10 cm from the tumor. A sigmoid colon colostomy was constructed in the left lower abdomen. Finally, the right ureter was reconstructed, and a double-J catheter was placed. The operation time was 6 h and 3 min, and the amount of blood loss was small. The postoperative course was uneventful, and the patient was discharged on the 25th postoperative day.

**Fig. 5:**
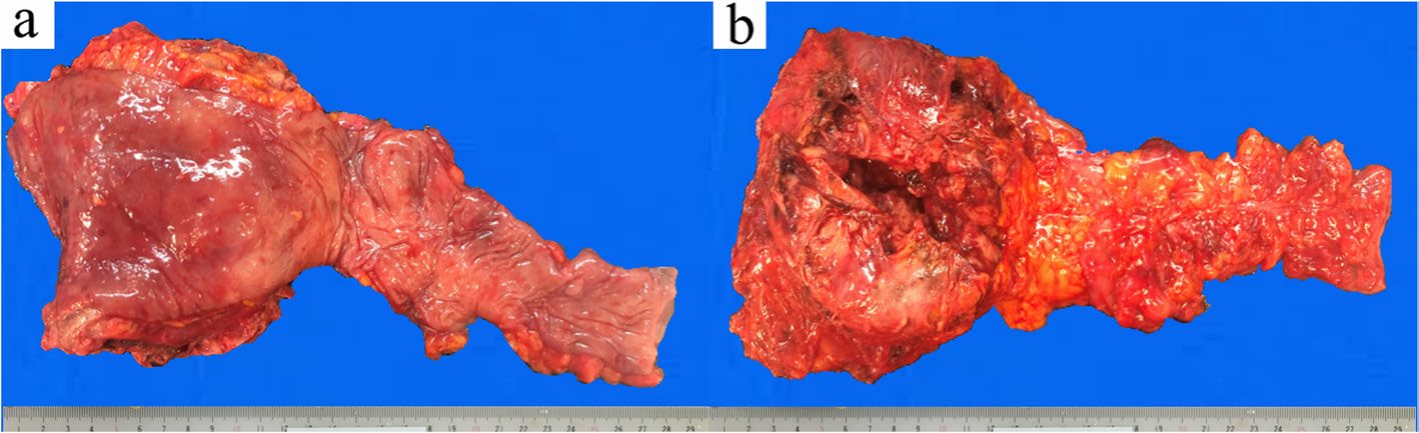
Macroscopic findings of the specimen. The mucosal aspect (**a**) and serous aspect (**b**) are presented. There was a 110 × 83 × 30 mm solid tumor with intraoperative damage and no obvious exposure to the mucosal surface

**Fig. 6:**
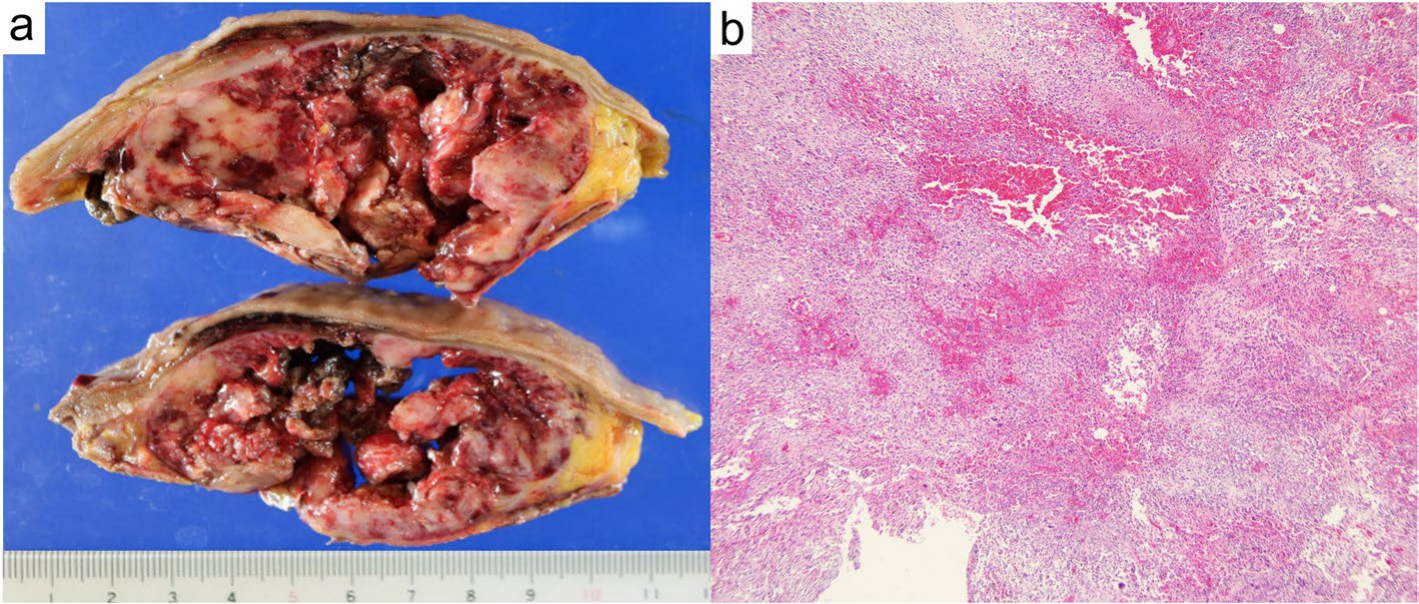
The cut surface of the tumor after fixation in formalin. The tumor consisted of a white solid with necrosis and hemorrhage in the center (**a**). Histopathologically, tumor cell growth was mainly observed in the muscularis propria to the subserosal layer of the rectum with inflammatory cell infiltration, hemorrhage, and necrotic tissue at the center (**b**, ×40 magnification).

**Fig. 7:**
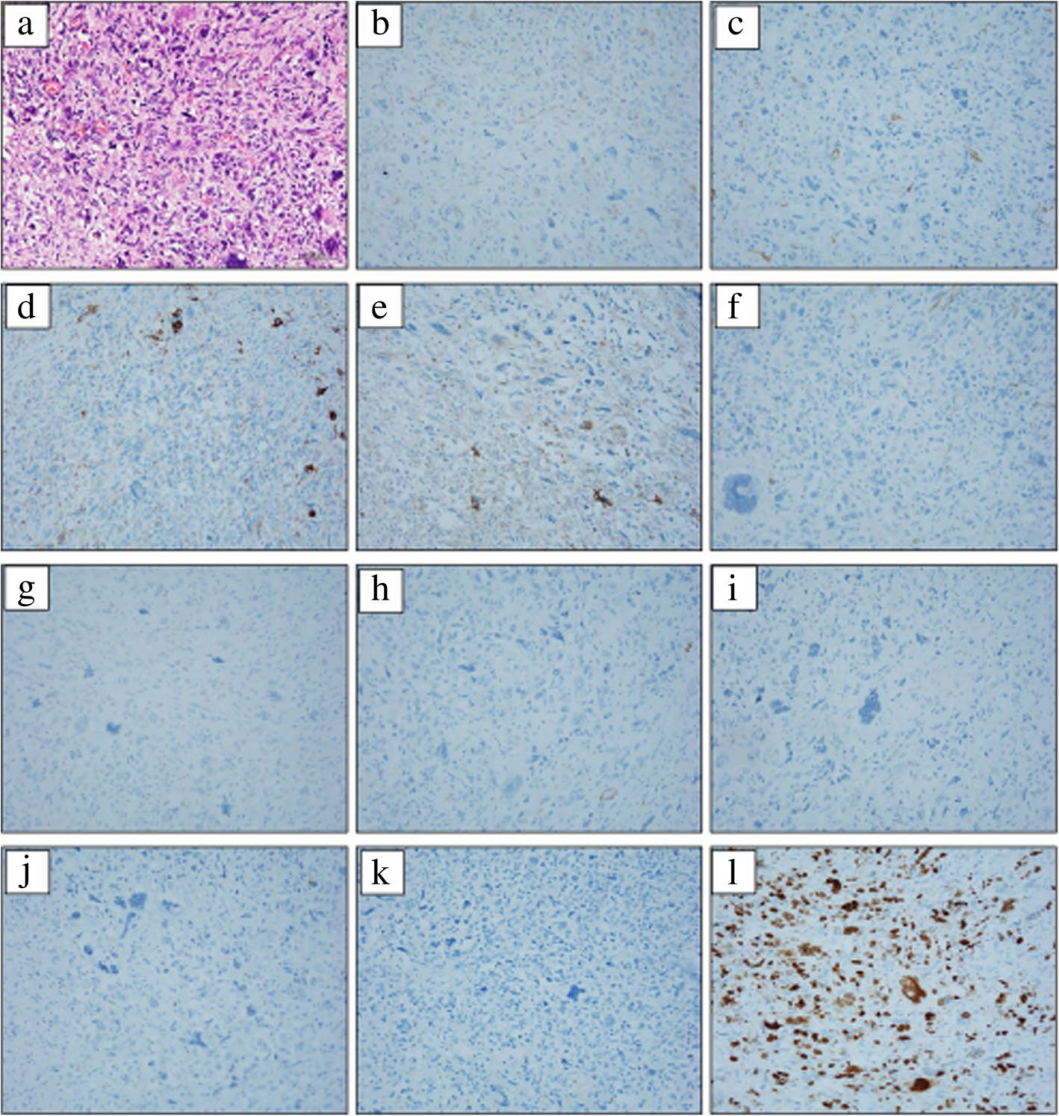
The histopathological findings and immunostaining. The tumor cells were composed of pleomorphic spindle cells and giant cells, and many typical and atypical mitotic figures were observed (**a**). On immunostaining, the tumor cells were focally positive for cluster of differentiation (CD) 117/KIT (**b**), α-smooth muscle actin (**c**), AE1/AE3 (**d**), and EMA (**e**) and negative for CD34 (**f**), DOG1 (**g**), h-caldesmon (**h**), desmin (**i**), S100 protein (**j**), and CD45 (**k**). Ki-67/MIB1 proliferation index was very high (**l**). (**a** ×100 magnification, **b**–**l** ×200 magnification)

Macroscopic observation of the resected specimen revealed that the rectum and sigmoid colon were 300 mm in length. On the serous aspect, there was a 110 × 83 × 30 mm solid tumor with intraoperative damage and no obvious exposure to the mucosal surface (Fig. 5 a and b). The cut surface was white solid with necrosis and hemorrhage in the center (Fig. 6a). Histopathologically, tumor cell growth was mainly observed in the muscularis propria to the subserosal layer of the rectum with inflammatory cell infiltration, hemorrhage, and necrotic tissue at the center (Fig. 6b). The tumor cells were composed of pleomorphic spindle cells and giant cells, and many typical and atypical mitotic figures were observed (Fig. 7a). On immunostaining, the tumor cells were focally positive for cluster of differentiation (CD) 117/KIT, α-smooth muscle actin, AE1/AE3, and EMA and negative for CD34, DOG1, h-caldesmon, desmin, S100 protein, and CD45. Ki-67/MIB1 proliferation index was very high (Fig. 7b-l). Immunostaining indicated that the tumor was composed of highly heterogeneous cells with no specific differentiation traits. Therefore, the diagnosis of UPS was made with the exclusion of diseases such as epithelial malignant tumor, gastrointestinal stromal tumor, melanoma, atypical lymphoma, and other undifferentiated/unclassified sarcoma. Lymphatic and venous invasion of the tumor was observed, but no lymph node metastasis was found.

**Fig. 8:**
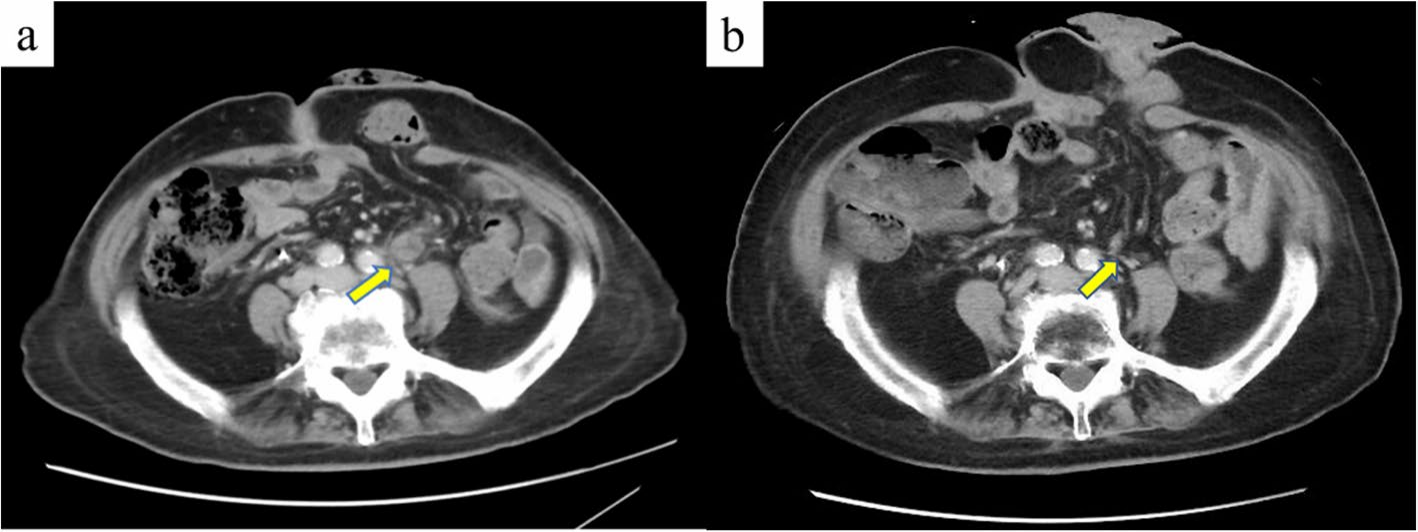
Contrast-enhanced computed tomography shows pelvic lymph node recurrence (**a**, indicated by an arrow). After radiotherapy, the metastatic lymph nodes disappeared (**b**, indicated by an arrow)

Contrast-enhanced CT performed 2 months after surgery showed bilateral pelvic lymph node enlargement and recurrence (Fig. 8a). Considering the patient’s age, we performed radiotherapy (50 Gy/25 Fr targeting the pelvic region). At present, 20 months have passed since the surgery, and 16 months have passed since the completion of radiotherapy. CT shows that the recurrent lymph nodes have disappeared, and no new distant metastasis has been observed (Fig. 8b).

## Discussion

UPS is a malignant soft tissue sarcoma (STS) that has been reclassified from MFH with the development of the pathological diagnosis. MFH was first documented as malignant histiocytoma and fibrous xanthoma by Ozello et al. in 1963 and was described as malignant fibrous xanthoma by O'Brien and Stout in the same group in the following year [3, 4]. In 1978, Weiss and Enzinger analyzed the clinicopathological features of 200 cases of MFH and established the concept of MFH. At that time, MFH was considered to be a malignant tumor derived from pleomorphic spindle cells that can differentiate into histiocytes and fibroblasts [2]. However, the accumulation of cases and pathological studies suggest that the histogenesis of MFH is undifferentiated mesenchymal cells. Furthermore, in recent years, STSs have been classified according to their tendency to differentiate, rather than the histogenesis. Therefore, the concept of MFH disappeared from the WHO disease classification in 2002 and 2013. In these WHO classifications, the major category of undifferentiated/unclassified sarcoma was created and further divided into five subtypes: undifferentiated round cell sarcoma, undifferentiated spindle cell sarcoma, undifferentiated pleomorphic sarcoma, undifferentiated epithelioid sarcoma, and undifferentiated sarcoma. MFH corresponds to the UPS [1]. UPS/MFH is frequently seen in individuals of 50–70 years of age, is more often seen in males, and is more frequent in Whites in comparison with Asians and Blacks. UPS/MFH occurs in the extremities and retroperitoneum, similarly to other soft tissue tumors, and rarely occurs in the gastrointestinal tract [5]. The clinical manifestations of colorectal UPS/MFH are nonspecific, and fever, abdominal pain, abdominal distension, weight loss, hemorrhage, and anorectal pain have been reported. The preoperative diagnosis of colorectal UPS/MFH is very challenging because of the rarity and variety of differential diseases [6]. CT images of UPS/MFH show it as a large soft tissue density mass that is relatively well-defined, segmental, and sometimes infiltrative. The center of the tumor frequently shows low attenuation, indicating necrosis, hemorrhage, and mucous degeneration [7]. MRI findings often demonstrate a heterogeneous signal on all sequences due to the various components. The solid components of the tumor exhibit enhancement after contrast agent administration [8].

The primary treatment for UPS is complete resection of the tumor, with the widest possible margins. Complete resection of the tumor has been reported to be closely related to the prognosis [9]. However, many STSs arising in the abdomen, including UPS, are very large and frequently invade vital organs at the time of the diagnosis. Therefore, the local recurrence rate and overall survival rate of STSs are inferior to those of extremity lesions because it is difficult to secure sufficient margins [10]. For UPS arising in the abdomen, it is more important to combine preoperative chemotherapy and radiotherapy in comparison with UPS that occurs on the surface of the body. Preoperative treatment of UPS must be discussed comprehensively with other STSs because of the rarity of the disease and the relative newness of the disease concept. The benefits of preoperative radiotherapy are a reduction in tumor size, preservation of the adjacent organs, and reduction of the risk of local recurrence. Several large retrospective analyses have reported that preoperative radiotherapy for retroperitoneal sarcoma contributes to the control of local recurrence and the prognosis [11, 12]. On the other hand, randomized STRASS trials have shown no clear benefit of preoperative radiotherapy [13]. In each trial, liposarcomas accounted for the majority of soft tissue sarcomas enrolled, and UPS accounted for a small proportion. Therefore, the efficacy of preoperative radiotherapy for UPS has not been established, but it may be effective only if the indications are carefully considered. Preoperative chemotherapy for retroperitoneal sarcoma is also controversial. Retrospective studies have indicated that it may have an adverse effect on the prognosis, presumably because of the characteristics of sarcoma, which is associated with a high risk of local recurrence. At present, a prospective, randomized phase III trial (NCT04031677) is underway in high-risk retroperitoneal sarcomas, and the results are awaited [14]. Cytotoxic therapy consisting of doxorubicin and ifosfamide is recommended for unresectable STS with distant metastasis. Recent progress in the analysis of the molecular pathogenesis of the genome, the development of novel-targeted therapies, and the accumulation of cases have also clarified the treatment of each subtype of STS [15]. The multi-kinase inhibitor sunitinib has been reported to have some antitumor efficacy against previously treated UPS in a phase II study [16]. UPS showed the higher expression of genes related to antigen presentation and T-cell infiltration in comparison with other STSs. Therefore, meaningful responses to nivolumab-ipilimumab combination therapy and pembrolizumab therapy have been reported in pretreated UPS [17, 18]. The combination of immune checkpoint inhibitors and radiotherapy for UPS that was refractory to conventional chemotherapy achieved complete response (CR) in a case report [19]. Currently, the effects of preoperative radiotherapy and immune checkpoint inhibitor therapy on retroperitoneal UPS are being explored [20].

A search of PubMed revealed that 28 cases of MFH/UPS occurring in the colorectum and anus were reported in the relevant English literature. Including our case, a total of 29 cases were reviewed. The male to female ratio of incidence was 20:9. The average age of the patients was 58 years (12–85 years). It occurred in all sites of the colorectum and anus, and this was the eighth case in the anorectal region. All patients were symptomatic, and the most common symptoms were abdominal pain, abdominal mass, abdominal distension, bloody stool, and diarrhea. The median diameter of the tumor was 7.2 cm (1.7–19 cm). All cases were treated by surgery with the exception of one autopsy case. Almost all surgery was performed by laparotomy, probably due to the large size of the tumor at the diagnosis. Our case is the first report of laparoscopic surgery for MFH/UPS in the colorectum and anus. Adjuvant chemotherapy was administered in 4 cases, adjuvant radiotherapy was administered in 3 cases, and adjuvant chemoradiotherapy was administered in 1 case; however, no patients had received neoadjuvant therapy. Among the nine patients with local or distant recurrence, mortality was reported in all but our case (Table 1).

**Table 1:** Cases of MFH/UPS in the colorectum and anus

Author	Age	Sex	Site	Size (cm)	Symptom	Primary treatment	Adjuvant	Recurrence	Prognosis (month)
Verma P. 1979 [21]	38	M	Rectum	12	Abdominal pain	Surgery (laparotomy)	No	No	Alive 14 M
Sewell R. 1980 [22]	74	M	Transverse	8.5 × 5 ×5	Anorexia, diarrhea	Surgery (laparotomy)	No	No	Alive 12 M
Levinson M. M. 1981 [23]	17	M	Transverse, rectosigmoid	10, 8.3 × 2	Abdominal pain, fever	Surgery (laparotomy)	No	ND	ND
Waxman M. 1983 [24]	52	F	Sigmoid	7.5 × 6	Abdominal pain	Surgery (laparotomy)	No	Yes (local)	Dead 9M
Rubbini M. 1983 [25]	60	M	Sigmoid	7	Bloody stool	Surgery (laparotomy)	Chemotherapy	Yes (liver, lymph node)	Dead 26 M
Spagnoli L. G. 1984 [26]	52	F	Anorectal	2.6 × 1.6 × 1	Bloody stool	Surgery (laparotomy)	No	Yes (lung, local)	Dead 24 M
Kukora J. S. 1985 [27]	73	M	Transverse	2.5 × 2	Abdominal pain, constipation	Surgery (laparotomy)	ND	No	Alive 48 M
Baratz M. 1986 [28]	73	M	Transverse	15 × 7 × 4, 8 × 4 × 1	Anorexia, anemia	Surgery (laparotomy)	No	No	Alive 6 M
Satake T. 1988 [29]	62	M	Ascending, transverse	17 × 10 × 8, 19 × 7 × 7	Abdominal mass	No (autopsy)	ND	ND	ND
Flood H. D. 1989 [30]	41	M	Anal canal	6	Abdominal mass	Surgery (laparotomy)	Radiotherapy	No	Alive 16 M
Katz R. N. 1990 [31]	62	F	Cecum	2 × 1.8 × 1.1	Abdominal pain	Surgery (laparotomy)	No	No	Alive 3 M
Murata I. 1993 [32]	50	M	Ascending	9.65 × 6.0 × 5.0	Abdominal distention, anorexia	Surgery (laparotomy)	Chemotherapy	No	Alive 10 M
Huang Z. 1993 [33]	12	M	Ascending	3.5	Abdominal pain	Surgery (laparotomy)	No	No	Alive 16 M
Makino M. 1994 [34]	72	M	Transverse	7.5 × 5.0	Abdominal pain	Surgery (laparotomy)	No	Yes (peritoneum)	Dead 4 M
Hiraoka N. 1997 [35]	64	M	Cecum	4 × 5 × 3	Abdominal distention	Surgery (laparotomy)	Chemotherapy	Yes (lymph node)	Dead 4 M
Kawashima H. 1997 [36]	50	F	Descending	4 × 3.2	Abdominal pain, diarrhea	Surgery (laparotomy)	No	No	Alive 84 M
Udaka T. 1999 [37]	47	M	Ascending	7 × 5 × 4	Abdominal mass	Surgery (laparotomy)	No	No	Alive 13 M
Singh D. R. 1999 [38]	55	M	Rectum	4 × 2.5	Tenesmus, perineal pain	Surgery (laparotomy)	Chemoradiotherapy	No	Alive 46 M
Okubo H. 2005 [39]	66	M	Ascending	14.5 × 8.0 × 4.5	Abdominal pain	Surgery (laparotomy)	No	No	Alive 33 M
Gupta C. 2006 [40]	46	F	Cecum, ascending	17	Abdominal distention, anorexia	Surgery (laparotomy)	No	No	Alive 36 M
Fu D. L. 2007 [41]	70	M	Cecum	12 × 10	Abdominal pain	Surgery (laparotomy)	No	Yes (lung)	Dead 1 M
Bosmans B. 2007 [42]	73	M	Sigmoid	3.2	Anemia	Surgery (laparotomy)	No	No	Alive 22 M
Kim B. G. 2008 [43]	63	F	Anal canal	1.7 × 1.3 × 0.3	Bloody stool, anal mass	Surgery (TAE)	Radiotherapy	No	Alive 15 M
Azizi R. 2011 [44]	80	M	Rectum	5 × 4 × 2.5	Rectal bleeding	Surgery (laparotomy)	No	ND	ND
Wang YJ 2012 [45]	55	M	Sigmoid	6	Abdominal pain	Surgery (laparotomy)	No	Yes (local)	Dead 5 M
Ji W. 2016 [46]	68	F	Ascending	8 × 6	Fever	Surgery (laparotomy)	Radiotherapy	Yes (local)	Dead 60 M
Kazama 2019 [47]	50	M	Ascending	7.2 × 6.0	Abdominal pain, numbness	Surgery (laparotomy)	Chemotherapy	No	Alive 6 M
Han X. 2022 [48]	65	F	Descending	10 × 8 × 5	Fever, fatigue	Surgery (laparotomy)	No	No	Alive 12 M
Our case 2022	85	F	Rectum	11 × 8.3 × 3	Anal pain	Surgery (laparoscopy)	No	Yes (lymph node)	Alive 12 M

*MFH* malignant fibrous histiocytoma, *UPS* undifferentiated pleomorphic sarcoma, *ND* not described

In our case, the resection of other pelvic organs should have been considered to achieve complete resection of the tumor. However, extended surgery is controversial because it is expected to impair quality of life. Preoperative treatment could have been considered if the pathological diagnosis had been obtained preoperatively. With the accumulation of evidence, preoperative treatment with a combination of radiotherapy, cytotoxic chemotherapy, immune checkpoint inhibitors, and molecular targeted agents may be performed in cases similar to ours.

## Conclusions

We reported a case of UPS arising in the rectum. The surgical procedure for UPS should be carefully selected because complete removal of the tumor is desirable. Indications for preoperative chemotherapy and radiotherapy should also be considered.

## Data Availability

Data sharing does not apply to this article as no datasets were generated or analyzed during the current study.

## Abbreviations

UPS: Undifferentiated pleomorphic sarcoma
MFH: Malignant fibrous histiocytoma
WHO: World Health Organization
CT: Computed tomography
MRI: Magnetic resonance imaging
CD: Cluster of differentiation
STS: Soft tissue sarcoma
